# Respiratory patterns and physical fitness in healthy adults: a cross-sectional study

**DOI:** 10.1186/s12889-024-17687-8

**Published:** 2024-01-19

**Authors:** Wen-Ming Liang, Yu-Xuan Ji, Jing Xiao, Inga Truskauskaitė, Adomas Hendrixson, Zhen-Min Bai, Osvaldas Ruksenas

**Affiliations:** 1 Department of Physiotherapy and Rehabilitation, Xiyuan Hospital, Chinese Academy of Chinese Medical Sciences, Beijing, China; 2 Life Sciences Center, Vilnius University, Vilnius, Lithuania; 3 Institute of Psychology, Vilnius University, Vilnius, Lithuania; 4 Faculty of Medicine, Vilnius University, Vilnius, Lithuania; 5 Department of Sports Rehabilitation, School of Sports Medicine and Rehabilitation, Beijing Sport University, Beijing, China

**Keywords:** Respiration rate, Inhalation duration, Exhalation duration, Inhalation exhalation ratio, Muscle performance, Reaction time, Vertical jump height

## Abstract

**Background:**

The altered respiratory patterns have a significant impact on our health. However, the links between respiration patterns during spontaneous breathing and physical fitness remain unknown. Therefore, we sought to examine how the respiratory pattern during spontaneous breathing interacts with physical fitness.

**Methods:**

A total of 610 participants (aged 20-59 years) were enrolled; 163 men (age = 41 ± 11) and 401 women (age = 42 ± 9) were included for analysis. The parameters of the respiration pattern were respiration rate (RR) and inhalation/exhalation (I/E) ratio. The physical fitness components were body size, visuomotor reaction time, balance, flexibility, hand grip strength, back extension strength, vertical jump height, number of push-ups, number of sit-ups, and the maximum rate of oxygen consumption. The data were analyzed separately for two gender groups. Participants within each gender group were further divided into two age categories (young: 20−39 years, middle-aged: 40−59 years) for the analysis, and both correlational and comparative tests were used to solidify the results.

**Results:**

Neither RRs nor the I/E ratios were substantially correlated with physical fitness in women. In addition, the I/E ratios showed no significant correlation with physical fitness in young men, while the results from correlational and comparative tests were inconsistent in middle-aged men. Consistently, men with lower RRs exhibited significantly shorter visuomotor reaction times in two age groups, and demonstrated significantly higher vertical jump heights in the middle-aged group.

**Conclusions:**

In women, respiratory patterns were not correlated with physical fitness. The relationship between middle-aged men’s I/E ratios and their physical fitness warrants further investigation. Men with lower RRs may have better visual-motor coordination and/or sustained attention, while middle-aged men with lower RRs may also have greater leg explosive power and neuromuscular coordination, which should be considered for physical assessment and health improvement.

## Introduction

Maintaining good physical health is essential for overall well-being and ensuring a high quality of life. A key indicator of physical health is physical fitness [1], which encompasses both health-related and skill-related fitness. Health-related fitness includes muscular strength, muscular endurance, flexibility, cardiorespiratory endurance, and body composition; skill-related fitness includes speed, power, agility, balance, coordination, and reaction time [2]. Thorough physical fitness tests provide a comprehensive assessment of one's overall physical well-being. Furthermore, respiration plays a vital role in our health, as breathing patterns have a profound impact not only on ventilation effectiveness but also on motor, cardiovascular, and autonomic nervous functions [3–5]. Therefore, the interaction between respiration patterns and physical fitness deserves attention.

The pattern of respiration includes a range of parameters, including rate, inhalation and exhalation duration, tidal volume, and depth [6]. Among these parameters, respiration rate (RR) and the ratio of inhalation to exhalation duration (I/E ratio) are particularly important. The RR serves as a crucial indicator of an individual's health status and is highly responsive to various stressors, including cognitive load, physical exertion, and exercise-induced fatigue [7]. A systematic review study summarized that intentionally reducing the RR yielded positive effects, such as enhanced ease, comfort, relaxation, and positive energy, and reduced feelings of anxiety, dejection, anger, hostility, and confusion [8]. One month of slow breathing training with a rate of 6 reps/min, as compared to the rate of 15 reps/min, improved exercise performance in chronic heart failure patients. Specifically, there were significant enhancements in the load reached and oxygen consumption during peak exercise [9]. In individuals with isolated systolic hypertension, eight weeks of slow-loaded breathing at a rate of 6 reps/min resulted in a significant improvement in arm exercise endurance [10]. In healthy subjects, engaging in 18 minutes of slow breathing led to an immediate reduction in visual reaction time, suggesting that slow breathing enhances the capacity for information processing and response inhibition [11]. On the other hand, fast breathing is known to enhance ventilation and elevate pH levels [12]. The elevation of pH levels enhanced Ca^2+^ and Na^+^ currents, reduced the action potential threshold, and shortened the refractory periods of action potentials, thereby promoting muscle contraction [13, 14]. Studies have demonstrated that intentionally induced hyperventilation as a strategy can improve repeated sprint performance, and pre-exercise hyperventilation can significantly enhance performance in the 50-meter front crawl [4, 5]. It appears that an intentionally slowed breathing rate can improve mental state and physical condition (e.g., higher visuomotor reaction speed and better muscle endurance), while purposefully fast breathing can enhance short-term explosive muscle strength. In addition to RR, the processes of inhalation and exhalation have distinct effects on the autonomic nervous system. During exhalation, the increased pressure in the chest cavity raises blood pressure [15], which activates the aortic arch baroreceptor and increases the stimulation of the nucleus tractus solitarius. This, in turn, leads to increased excitation of parasympathetic efferent signals. Conversely, the effect of inhalation on sympathetic activity is vice versa [16–18]. Thus, the I/E ratio received attention in the psychological research. Experiments have shown that deliberately decreased I/E ratio enhance the cardiac vagal tone [19]. Participants experienced increased relaxation, positive energy, reduced stress, and heightened mindfulness when adopting a breathing pattern with a low I/E ratio compared to a high ratio [20]. These findings indicate that lowering the I/E ratio is able to promote parasympathetic nervous activity.

However, the relationship between spontaneous respiratory patterns and physical fitness remains unclear, and clarifying the relationship can offer healthcare professionals more information for physical assessment and health improvement. Therefore, the objective of the current study was to examine the relationship between respiratory patterns (RR and I/E ratio) and physical fitness components (muscle endurance, muscle explosive power, balance, flexibility, visuomotor reaction time, and cardiopulmonary endurance). The goal was to determine whether people with lower or higher RRs and/or I/E ratios exhibit differences in physical fitness.

During spontaneous breathing, the diaphragm muscle performs 60–80% of the inspiratory work [21]. A slower breathing rate necessitates a larger tidal volume to maintain normal ventilation [22], which should increase the workload of the diaphragm muscle. The diaphragm is one of the main core muscles responsible for trunk stability [23], and enhanced trunk stability facilitates improved physical performance [24, 25]. In conjunction with the findings cited in the second paragraph, we hypothesize that participants with lower respiratory rates (RRs) will demonstrate better physical fitness.

As for the I/E ratio, it is influenced by many factors. During inhalation, the diaphragm and external intercostal muscles contract to enlarge the thoracic cavity, which causes a decrease in intra-thoracic pressure and enables air to enter the lungs [12]. Thus, the strength of the diaphragm and external intercostal muscles likely impacts the duration of inhalation. In a resting state, individuals with higher inspiratory muscle strength should have a larger inspiratory movement and, consequently, a longer inhalation duration. Notably, improved inspiratory muscle strength is associated with enhanced physical performance (e.g., 800-meter run, peripheral muscle strength) [26, 27]. Normal exhalation represents a passive process as it relies on the elastic recoil of the muscles and lungs [28]. Therefore, inhalation duration should be the main factor impacting the I/E ratio, leading to the hypothesis that people with a higher I/E ratio would demonstrate better muscle performance. However, studies referenced in the second paragraph indicate that participants experienced increased relaxation when taking a breathing pattern with a low I/E ratio. Given that chronic stress significantly slows task response [29], participants with lower I/E ratios may exhibit faster reaction speeds.

## Methods

### Trial design and participants

**Fig. 1:**
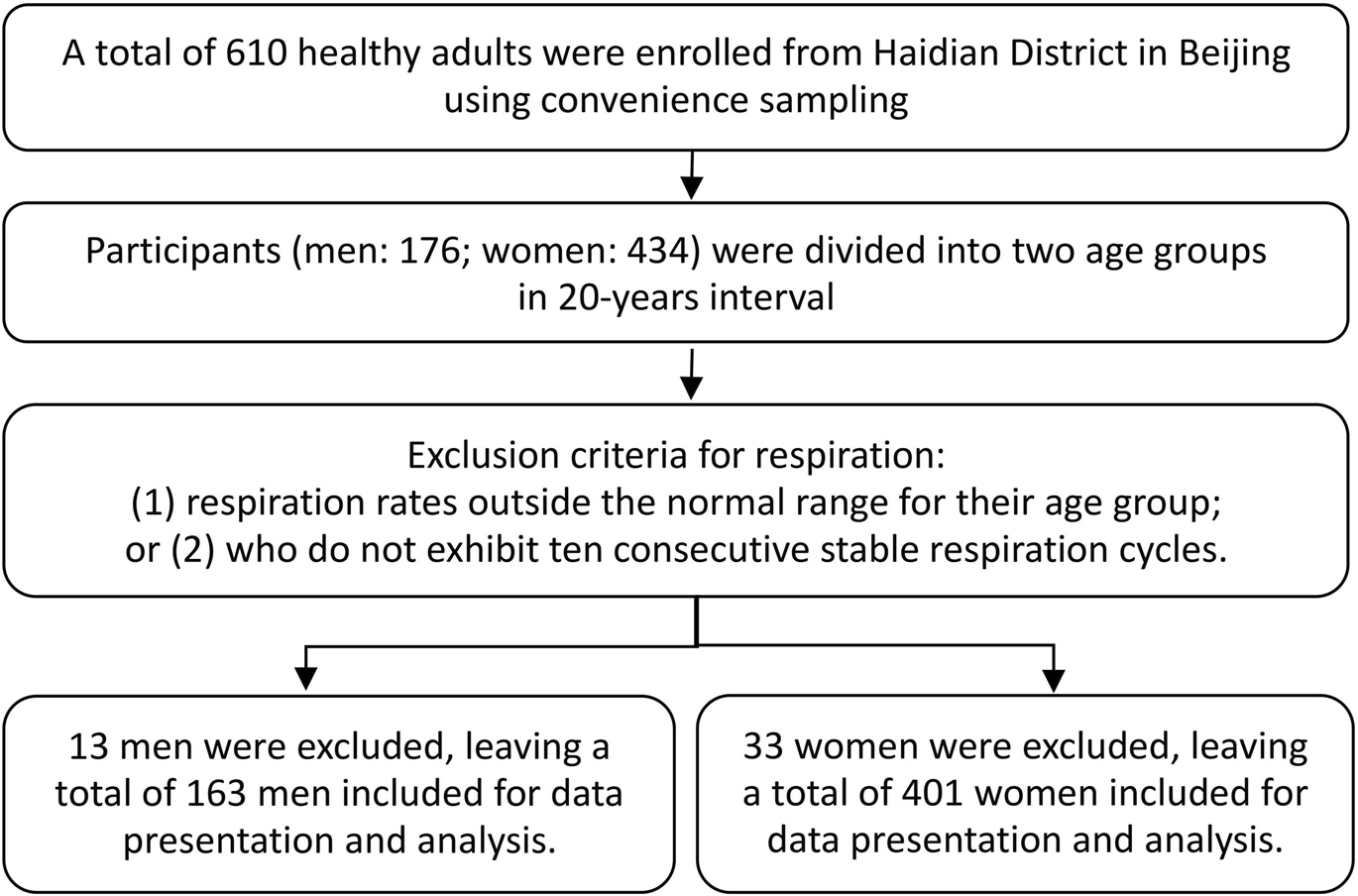
Flowchart of study participants

A total of 610 healthy adults (aged 20–59) were enrolled through convenience sampling from six communities in Haidian District in Beijing. The inclusion criteria for participants were as follows: 20–59 years old, capable of understanding and responding to the interview questions, having completed the Physical Activity Readiness Questionnaire and meeting all requirements, and providing written informed consent. Exclusion criteria: pregnancy or lactation; the presence of a mental illness; recent or ongoing acute diseases without physical recovery; consumption of coffee or tea within 2 hours prior to the tests; having a RR exceeding ± 2 times the standard deviations from the average in their age group (subjects excluded if RR < mean – 2 × SD or RR > mean + 2 × SD); did not perform ten consecutively stable respiratory cycles from a two-minute respiration test. The final study sample consisted of 564 participants, 163 men (age = 41 ± 11, BMI = 25.8 ± 4.1) and 401 women (age: 42 ± 9, BMI: 23.3 ± 3.5), as shown in Fig. 1.

The Research Ethics Committee of Beijing Sport University approved the present study (Approval number: 2021079H), and all participants were informed of the risks of the tests prior to signing the informed consent document.

### Data collection and processing

#### Respiratory movements testing and data processing

Respiration was recorded by a respiration belt (Vernier, Beaverton, OR, USA) which is a strap of fabric with a resistive stretch sensor embedded into it and provides ground truth respiration rate signals [30]. Prior to the test, participants were instructed to remain seated quietly for 5 minutes. Then, participants stood up, the experimenter tied the belt at the level of the xiphoid process of the participants until the light on the belt turned green according to the user's instructions. Throughout the two-minutes test, the participants were asked to watch a neutral video featuring slow-swimming fishes in the sea. The video was displayed on a Xiaomi Pad (11 inches, Xiaomi, Beijing, China), positioned in front of the participant's face at a distance ranging from 50 to 80 cm.

Different authors employed various approaches to determine the number of respiratory cycles for analysis, ranging from three satisfactory readings to six minutes of breathing cycles [31–33]. We observed that the respiration waves became more regular after 30 seconds from the beginning. Consequently, we chose ten consecutive respiration cycles that demonstrated consistent stability, minimal motion artifact, and baseline wander after the initial 30 seconds of the testing period.

**Fig. 2:**
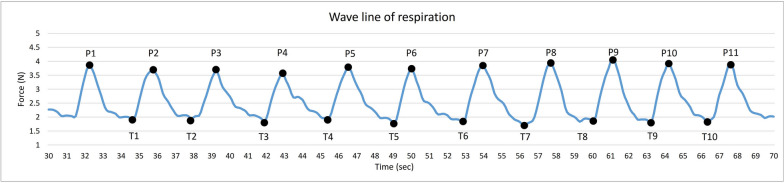
Wave lines of respiration. P, peak; T, trough. Sec, second; N, Newton

A Matlab App Designer program (Matlab 2022a, Mathworks, Natick, MA, USA) was used to process the raw data and target the maximum peaks (indicating the end of inspiration) and minimum troughs (indicating the end of expiration). Safeguards were implemented to decrease the chance of flagging false minimum and maximum values in data with higher noises. As shown in Fig. 2, the inhalation duration (ID) was calculated by subtracting the time at the peak from the preceding trough and then averaging across all ten summed IDs. Exhalation duration (ED) was calculated by subtracting the time of trough from the peak time that preceded it and averaging across all ten summed EDs. The I/E ratio was calculated by dividing the ID by the ED. The RR was determined as 60 seconds divided by the time used for one respiration cycle, which was calculated from the time of the 11th peak minus the time of the 1st peak, divided by 10 (RR = 60/(P11 − P1)/10).

#### Physical fitness testing

All physical fitness tests were performed using the Jianmin electronic physical fitness assessment system (Xindonghuateng Sports Equipment Co., Ltd., Beijing, China), which is approved by the Sports Equipment Approval Committee of the General Administration of Sport in China. The testing protocol was developed and executed in accordance with the manual National Physical Fitness Testing and Evaluation [34].

#### Body size tests

Height measurements were taken without shoes using an electronic body height measuring instrument (Jianmin GMCS-SGJ3, Xindonghuateng, Beijing, China) with an accuracy of 1 cm. Weight measurements were taken on an electronic weighing scale (Jianmin GMCS-RCS3), with an accuracy of 0.1 kg. Body fat percentage was assessed using a body composition analyzer (Jianmin GMCS-TZL3). Participants stood barefoot on two electrode plates while holding two electrode handles for one minute. The accuracy of the measurement was 0.1 kg. Waist and hip circumference were measured using an electronic circumference measuring ruler (Jianmin GMCS-WD3) at the navel height and the widest part of the buttocks. The accuracy of these measurements was 0.1 cm.

**Fig. 3:**
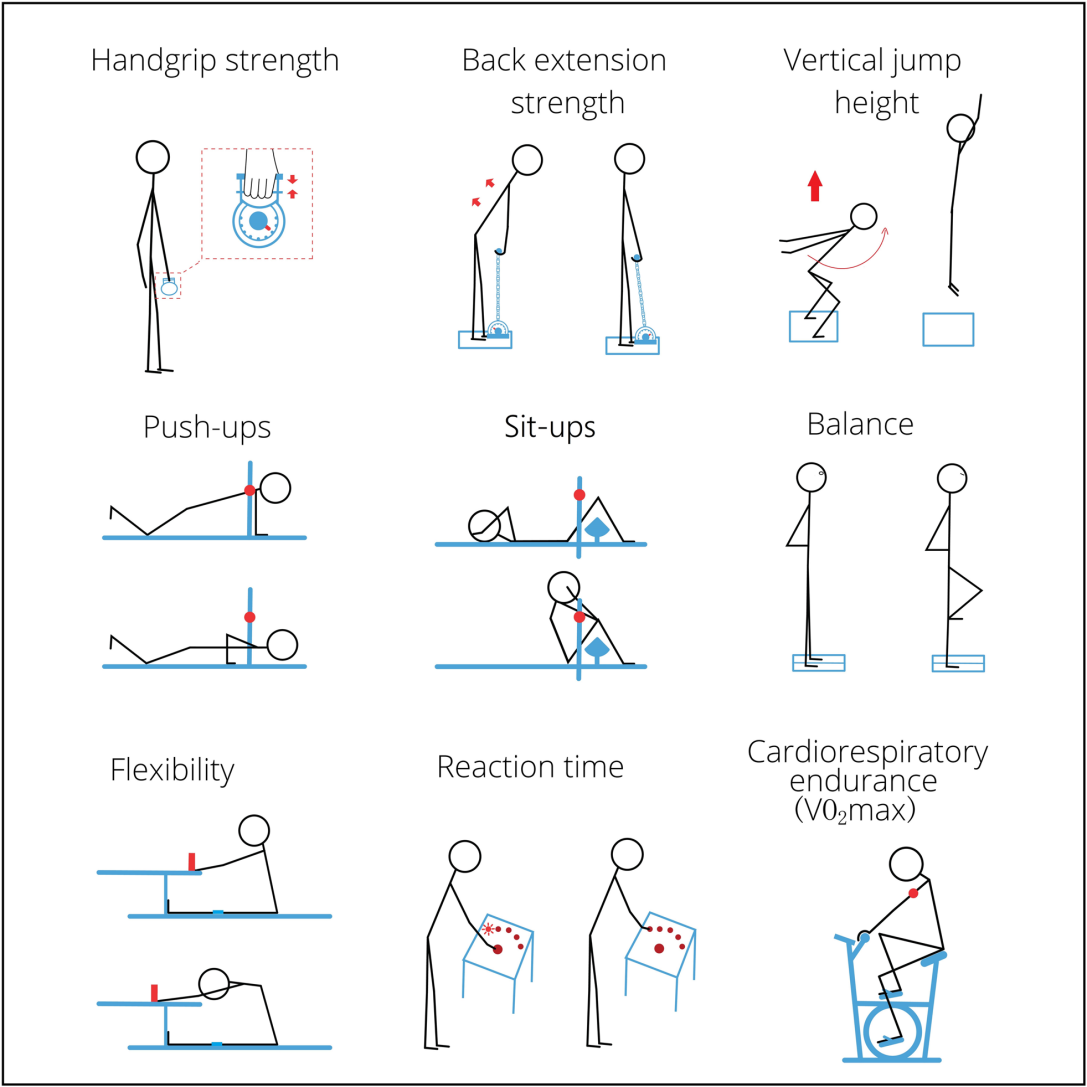
An illustration of the tests of physical fitness. This figure was adapted from our previous publication [35]

For a clearer illustration of tests on muscular strength, muscular power, muscular endurance, balance, flexibility, visuomotor reaction time, and cardiorespiratory endurance, we have provided Fig. 3 below.

#### Muscular strength tests

Before conducting the strength test, participants received a detailed explanation of the testing procedures and were advised to engage in a five-minute warm-up. The warm-up routine included 30 seconds of jogging in place, dynamic stretching for the main joints (e.g., neck, shoulders, wrists, back, hips, knees) for two minutes, and performing the movements to be tested for two minutes (without equipment, with participants deciding on the movements and repetitions). A physical therapist stood by to provide assistance and answer questions. Subsequently, participants stood upright and held a handgrip dynamometer (Jianmin GMCS-WCS3) in their dominant hand, positioned approximately 10-20 cm away from their thigh, with the palm facing inward. Following a two-minute rest from the handgrip strength test, participants proceeded to perform a back extension test using another dynamometer (Jianmin GMCS-BLJ3). This dynamometer consisted of a standing plate, a hand-holding bar, and a chain connecting the plate and bar. Participants stood on the plate with their hands hanging down and fingers extended in front of their thighs. The experimenter adjusted the chain length and positioned the bar at the height of the participants' middle fingertips. Subjects flexed their hips, maintaining straight arms, legs, and trunk, and then gradually lifted the bar with maximal effort. Each muscular strength test was conducted twice, and the higher value was recorded with a precision of 0.1 kg.

#### Muscular power tests

The countermovement jump with arm swing was utilized to assess vertical jump height. Participants stood on a timing mat (Jianmin GMCS-ZTJ3), which featured an integrated calculator capable of measuring the time spent in the air and calculating the corresponding jump height. Each participant performed two jumps, and the highest recorded value was documented with a precision of 0.1 cm.

#### Muscular endurance tests

Participants positioned themselves facing the floor, distributing their weight between their straight hands and legs, utilizing push-up counting equipment (Jianmin GMCS-FWC3). An assistant adjusted two laser detectors to align with the participants' shoulder height. Subsequently, participants engaged in the standard push-up exercise for one minute. The system accurately recorded the instances when participants elevated their trunks and reached the designated shoulder height. Similarly, the number of sit-ups completed within one minute was recorded using sit-up counting equipment (Jianmin GMCS-YWQZ3), employing a similar methodology.

#### Balance test

Participants stood with their arms akimbo at a balance testing equipment (Jianmin GMCS-DJZL3). They closed their eyes and raised one foot off the ground when prepared. The system initiated the recording when the participant lifted their foot and ceased recording when the other foot moved away. The precision of the recorded standing time was 0.1 seconds.

#### Flexibility test

Participants sat on the equipment (Jianmin GMCS-TQQ3) without shoes and straightened their legs against a designated box. The experimenter secured their knees to maintain a straight alignment. With their palms facing downwards, participants reached forward along the measuring bar, striving to extend as far as they could. The test was conducted two times, and the greater distance achieved was recorded with a precision of 0.1 cm.

#### Visuomotor reaction time test

A visuomotor reaction time testing panel (Jianmin GMCS-FYS3) consists of a starting button and five signal buttons. Participants stood in front of the panel, placing their dominant hand on the starting button. During each trial, one of the signal buttons randomly illuminated, and participants were required to quickly press the illuminated button. Each round consists of five trials, and the average time taken to respond in those trials was calculated as the final result. The participants engaged in two rounds, and the shorter response time was recorded with a precision of 1 millisecond (ms).

#### Cardiorespiratory endurance test

To estimate the maximal oxygen consumption (VO2max), the YMCA submaximal cycle ergometer (Jianmin GMCS-GLC3) was employed. Following a five-minute period of rest, participants positioned themselves on the cycle ergometer while wearing an optical heart rate sensor (Polar OH1, Kempele, Finland) on their upper arm. The heart rate sensor was connected to the physical fitness assessment system via Bluetooth, enabling the system to calculate heart rate and introduce workloads accordingly. The test lasted for a total of seven minutes, with 30 seconds allocated for establishing the baseline heart rate, three minutes each for the first and second stage heart rates, and a final 30 seconds for cooling down. For a detailed explanation of the calculation method, please refer to the study conducted by Nuria Garatachea et al. [36].

### Normalization of muscular performance

As grip strength, back extension strength, push-ups, sit-ups, balance were influenced by body weight, and studies recommended to normalized the muscular performance with weight using the equation:

1 $$\mathrm{Pn }=\mathrm{ P}/{\text{Mb}}$$

where P is the outcome of muscular performance, Pn is the normalized outcome of muscular performance, M is the body mass, and b is the allometric value [37, 38]. The allometric values (b) for hand grip strength and back extension strength were 0.67, and for push-ups, sit-ups, and balance was −0.33 [38]. Visuomotor reaction time, vertical jump height, and flexibility were not influenced by body size and did not require normalization. Additionally, the outcome of cardiorespiratory endurance has already been adjusted for body size and does not require further normalization.

### Data analyses

Since the distributions of some parameters were skewed according to the Kolmogorov-Smirnov test, the Mann-Whitney U Test was conducted to compare two age groups (young: 20−39 years, middle-aged: 40−59 years), and the data were presented as the median and interquartile range (IQR). The Spearman correction test was employed for correlation tests. The linear regression method was used to generate residuals between age and the physical fitness components, for which it had a significant correlation with age. Additionally, to explore differences in physical fitness between groups with longer and shorter RR, ID, ED, and high and low inhalation/exhalation ratios, these four respiratory parameters were each bifurcated based on their median values. Given the observed correlation between RR and physical fitness, the K-means clustering method was applied to categorize participants into two clusters according to ID and ED. The physical fitness levels of these clusters were then compared. Due to the skewness in the distribution of certain respiratory parameters, the Mann-Whitney U Test was again employed for the comparisons of both median-split and clustered groups.

The primary analytical method is the correlation test, while the comparative test serves as a secondary approach. Based on the results of power analysis, to obtain a medium sized correlation (0.3), α = 0.05 (one tailed), power = 0.8) [39], 67 participants were needed. The significance level was set at *p* < 0.05. The strength of the effect size (Rho) from the Spearman correlation test was adopted as negligible (Rho < 0.20), weak (0.21 < Rho < 0.40), moderate (0.41 < Rho < 0.60), strong (0.61 < Rho < 0.80), and very strong (0.81 < Rho < 1.00) [40]. The effect sizes (r) from the Mann-Whitney U Test were derived from the z-values divided by the square root of the sample size [41], and it was referred to as small (d < 0.2), medium (0.2 < d < 0.5), and large (d > 0.5) according to other studies [42]. All data were calculated and analyzed using Excel 365 (Excel, Microsoft, Redmond, WA) and SPSS 20.0 (IBM Corp., Armonk, NY, USA). The power analysis was conducted with G*Power software.

## Results

### Data description and comparison of all parameters between age groups in men and women

The comparison of all study parameters across age groups is presented in Table 1 for men and in Table 2 for women. The results indicate that there are no significant differences in men’s RR, ID, ED, and I/E ratio between the young (aged 20-39 years) and middle-aged (aged 40-59 years) groups (Table 1). However, young men exhibited higher body height, weight, hip circumference, back extension strength, vertical jump height, number of sit-ups in one minute, and balance, while middle-aged men had longer reaction times.

**Table 1:** Data presentation and comparison between age groups in men (*n*=163)

**All parameters**	**Age**^**1**^	**n**^**1**^	**Median (IQR)**^**1**^	**Age**^**2**^	**n**^**2**^	**Median (IQR)**^**2**^	**z**	***p***	**r**
Age (year)	**20-39**	70	32.0 (10.0)	**40-59**	93	49.0 (10.5)	10.9	0.000	0.86
Height(cm)		70	176 (8.45)		93	172 (8.55)	2.95	0.003	0.23
Weight (kg)		70	80.4 (18.2)		93	73.9 (14.4)	2.31	0.021	0.18
BMI (kg/m^2^)		70	25.7 (5.29)		93	25.2 (4.47)	1.30	0.193	0.10
WC (cm)		67	89.8 (13.0)		90	92.0 (10.7)	1.36	0.173	0.11
HC (cm)		67	102 (9.80)		90	97.8 (9.38)	2.18	0.029	0.17
WH ratio		67	0.89 (0.09)		90	0.93 (0.07)	3.79	0.000	0.30
Body fat (%)		67	24.9 (7.32)		91	24.2 (6.90)	1.06	0.290	0.08
RR (rep/min)		70	15.2 (4.52)		93	17.1 (5.52)	1.22	0.224	0.10
ID (s)		70	1.53 (0.53)		93	1.41 (0.70)	1.27	0.205	0.10
ED (s)		70	2.19 (0.71)		93	2.03 (0.72)	1.05	0.292	0.08
I/E ratio		70	0.70 (0.23)		93	0.73 (0.18)	0.14	0.891	0.01
Grip strength (kg)		69	43.5 (11.0)		93	41.5 (9.80)	1.65	0.099	0.13
**Back strength (kg)**		**69**	**120 (35.2)**		**93**	**111 (41.0)**	**2.32**	**0.021**	**0.18**
**Jump height (cm)**		**70**	**37.0 (12.5)**		**93**	**27.5 (12.5)**	**5.57**	**0.000**	**0.44**
Push-ups (rep/min)		65	20.0 (19.5)		79	18.0 (15.0)	1.38	0.168	0.11
**Sit-ups (rep/min)**		**67**	**28.0 (11.0)**		**75**	**20.0 (11.0)**	**3.65**	**0.000**	**0.31**
**Balance (s)**		**70**	**14.4 (20.9)**		**90**	**11.3 (13.5)**	**2.04**	**0.042**	**0.16**
Flexibility (cm)		69	2.90 (15.3)		89	3.30 (14.1)	0.59	0.556	0.05
**Reaction time (s)**		**68**	**0.52 (0.09)**		**85**	**0.58 (0.12)**	**3.84**	**0.000**	**0.31**
VO_2_ max (mL/(kg×min))		44	39.9 (10.8)		52	40.4 (12.6)	-0.31	0.760	0.03

*BMI* Body mass index, the weight in kilograms divided by the square of the height in meters, *WC* Waist circumference. *HC* Hip circumference, *WH ratio* Waist/hip circumference ratio, *ID* Inhalation duration, *ED* Exhalation duration, *I/E ratio* Inhalation exhalation ratio, *VO*_*2*_* max* The maximum rate of oxygen consumption, *N* Newton, kg Kilogram, *cm* Centimeter, *ml* Milliliter. Age^1^, n^1^, and Median (IQR)^1^ are for the young group (20–39 years), while age^2^, n^2^, and Median (IQR)^2^ for the middle-aged group (40-59 years).

**Table 2:** Data presentation and comparison between age groups in women (*n*=401)

**All parameters**	**Age**^**1**^	**n**^**1**^	**Median (IQR)**^**1**^	**Age**^**2**^	**n**^**2**^	**Median (IQR)**^**2**^	**z**	***p***	**r**
Age (year)	20-39	154	34 (8)	40-59	247	48 (10)	16.9	0.000	0.84
Height (cm)		154	161 (7.12)		247	161 (6.70)	0.37	0.710	0.02
Weight (kg)		154	58.3 (13.4)		247	61.1 (11.8)	1.85	0.065	0.09
BMI (kg/m^2^)		154	22.2 (5.15)		247	23.0 (4.11)	1.96	0.050	0.10
WC (cm)		151	73.3 (13.6)		245	77.5 (11.9)	3.15	0.002	0.16
HC (cm)		151	93.7 (9.50)		245	94.6 (9.05)	0.61	0.540	0.03
WH ratio		151	0.79 (0.08)		245	0.82 (0.08)	3.75	0.000	0.19
Body fat (%)		151	27.6 (9.07)		244	29.7 (6.79)	2.21	0.027	0.11
RR (rep/min)		154	17.2 (4.39)		247	17.1 (4.83)	0.68	0.494	0.03
ID (s)		152	1.40 (0.44)		247	1.38 (0.44)	0.30	0.760	0.02
ED (s)		152	2.08 (0.61)		247	2.15 (0.68)	1.32	0.188	0.07
I/E ratio		152	0.69 (0.18)		247	0.66 (0.17)	1.65	0.099	0.08
Grip strength (kg)		150	24.6 (6.60)		244	25.8 (7.25)	1.65	0.100	0.08
Back strength (kg)		153	62.3 (23.7)		241	66.5 (22.9)	2.15	0.031	0.11
Jump height (cm)		153	22.6 (6.80)		243	19.6 (6.50)	5.63	0.000	0.28
Push-ups (rep/min)		141	17.0 (14.5)		229	15.0 (14.0)	1.32	0.186	0.07
Sit-ups (rep/min)		141	23.0 (13.0)		218	17.0 (12.3)	5.12	0.000	0.27
Balance (s)		153	22.0 (25.5)		246	15.2 (21.9)	3.49	0.000	0.17
Flexibility (cm)		150	8.65 (13.9)		245	10.9 (12.8)	1.88	0.061	0.09
Reaction time (s)		148	0.57 (0.08)		245	0.60 (0.11)	3.72	0.000	0.19
VO_2_ max (mL/(kg×min))		116	41.5 (11.2)		192	34.3 (8.03)	6.51	0.000	0.37

*BMI* Body mass index, the weight in kilograms divided by the square of the height in meters, *WC* waist circumference, *HC* Hip circumference, *WH ratio* Waist/hip circumference ratio, *ID* inhalation duration, *ED* Exhalation duration, *I/E ratio* inhalation exhalation ratio, *VO*_*2*_* max* The maximum rate of oxygen consumption; Age in years; *N* Newton; *kg* Kilogram, *cm* Centimeter, *ml* Milliliter. Age^1^, n^1^, and Median (IQR)^1^ are for the young group (20–39 years), while age^2^, n^2^, and Median (IQR)^2^ for the middle-aged group (40-59 years)

As for women (Table 2), RR, ID, ED, and I/E ratio were also not significantly different between the young and middle-aged groups. But, waist circumference, waist-hip circumference, body fat percentage, back extension strength, and reaction time were higher in middle-aged group. At the same time, vertical jump height, number of sit-ups, balance, and VO_2_max were higher in young group.

### Correlation between respiration patterns and physical fitness in men and women

Since many physical fitness components were significantly different between the young and middle-aged groups, correlation analysis was conducted in these two age groups separately. For young men, there was no significant correlation between age and physical fitness components. Whereas, in the middle-aged group, age was significantly correlated with vertical jump height (*n* = 93, Rho = -0.451, *P* < 0.001), with sit-ups (*n* = 75, Rho = −0.272, *P* = 0.018), with balance (*n*=90, Rho = −0.405, *P* < 0.001), and reaction time (*n* = 85, Rho = 0.242, *P* = 0.025). Therefore, the generated residuals of these four performances were used for the correlational test.

The ID and ED were strongly correlated with RR since they were calculated from RR. However, RR was not correlated with the I/E ratio in the young group but had a weak and negative correlation in the middle-aged group. Regarding physical fitness, all respiration parameters were not correlated with body size (Table 3). Men’s RR had a weak and positive correlation with visuomotor reaction time from the two age groups, which indicated that men with lower RR might have a faster reaction speed. Additionally, for middle-aged men, their RR showed a weak and negative correlation with vertical jump height, suggesting that men aged 40−59 years with higher RR might have a lower jump height. For ID, it had a positive and weak correlation with the number of sit-ups in the young group and back extension strength in the middle-aged group. ED had a negative and weak correlation with visual reaction time in young group. I/E ratios had a weak and positive correlation with back extension strength and the number of sit-ups in middle-aged men, which indicated that men with longer inhalation than exhalation might have better strength in the back, abdominal, and hip flexor muscles.

**Table 3:** Correlation between respiration duration and physical fitness in men (*n*=163)

**Parameter (men)**	**Age group**^**1**^	**n**^**1**^	**RR**^**1**^	**ID**^**1**^	**ED**^**1**^	**I/E ratio**^**1**^	**Age group**^**2**^	**n**^**2**^	**RR**^**2**^	**ID**^**2**^	**ED**^**2**^	**I/E ratio**^**2**^
			Rho	Rho	Rho	Rho			Rho	Rho	Rho	Rho
Age (year)	20-39	70	0.100	-0.132	-0.030	-0.193	40-59	93	0.153	-0.089	-0.180	0.101
Height (cm)		70	-0.003	-0.109	0.047	-0.156		93	0.085	-0.054	-0.059	-0.038
Weight (kg)		70	-0.096	-0.039	0.087	-0.078		93	0.165	-0.172	-0.092	-0.128
BMI (kg/m^2^)		70	-0.063	-0.032	0.038	-0.028		93	0.120	-0.146	-0.053	-0.122
WC (cm)		70	-0.177	0.075	0.128	-0.019		90	0.171	-0.178	-0.105	-0.161
HC (cm)		67	-0.176	0.026	0.140	-0.050		90	0.096	-0.091	-0.068	-0.060
WH ratio		67	-0.123	0.077	0.088	-0.014		90	0.207	-0.188	-0.159	-0.120
Body fat (%)		67	-0.103	0.042	0.059	0.017		91	0.137	-0.164	-0.061	-0.140
Grip (kg)		69	0.021	0.064	0.015	0.033		93	-0.169	0.149	0.142	0.106
Back (kg)		69	-0.151	0.171	0.203	-0.038		93	-0.192	.229*	0.112	.221*
Jump (cm)		70	-0.157	0.210	0.116	0.094		93	-.239*	.189	.227*	0.033
Push-ups (rep/min)		65	0.068	0.060	-0.090	0.132		79	-0.175	0.177	0.109	0.142
Sit-ups (rep/min)		67	-0.240	.242*	0.157	0.113		75	-0.120	0.200	0.030	.245*
Balance (s)		70	-0.123	0.147	0.071	0.003		90	-0.090	0.054	0.124	-0.082
Flexibility (cm)		69	-0.092	0.068	0.104	0.051		89	-0.128	0.175	0.028	0.194
Reaction time (s)		68	.278*	-0.189	-.291*	0.109		85	.246*	-.240*	-0.170	-0.170
VO_2_ max (mL/(kg×min))		44	0.025	0.039	0.021	0.072		55	-0.069	0.054	0.072	-0.063

*BMI* Body mass index, the weight in kilograms divided by the square of the height in meters, *WC* Waist circumference, *HC* Hip circumference, *WH ratio* Waist/hip circumference ratio, *ID* Inhalation duration, *ED* Exhalation duration, *I/E ratio* Inhalation exhalation ratio, *VO*_*2*_* max* The maximum rate of oxygen consumption, *N* Newton, *kg* Kilogram, *cm* Centimeter, *ml* Milliliter. Age^1^, n^1^, RR^1^,ID^1^, ED^1^, and I/E ratio^1^ are for the young group (20-39 years), while age^2^, n^2^, RR^2^, ID^2^, ED^2^, and I/E ratio^2^ for the middle-aged group (40-59 years). * *p* < 0.05.

Regarding women, as shown in Table 4, the respiration patterns were not correlated with all physical fitness components. Besides, the ID and ED were strongly correlated with respiration rate, while RR was not correlated with the I/E ratio.

**Table 4:** Correlation between respiration duration and physical fitness in women (*n*=401)

**Parameter** **(women)**	**Age** **Group**^**1**^	**n**^**1**^	**RR**^**1**^	**ID**^**1**^	**ED**^**1**^	**I/E ratio**^**1**^	**Age** **Group**^**2**^	**n**^**2**^	**RR**^**2**^	**ID**^**2**^	**ED**^**2**^	**I/E ratio**^**2**^
			Rho	Rho	Rho	Rho			Rho	Rho	Rho	Rho
Age (year)	20-39	152	0.145	-0.105	-0.068	-0.068	40-59	247	0.078	-0.045	-0.078	0.020
Height (cm)		152	-0.046	0.005	0.053	0.014		247	-0.062	0.085	0.045	0.046
Weight (kg)		152	0.005	-0.019	0.053	-0.049		247	-0.005	-0.021	0.023	-0.048
BMI (kg/m^2^)		152	0.001	0.005	0.057	-0.050		247	0.049	-0.085	-0.020	-0.068
WC (cm)		149	0.022	-0.040	0.043	-0.060		245	0.059	-0.075	-0.030	-0.052
HC (cm)		149	0.013	-0.012	0.015	0.007		245	0.023	-0.055	0.014	-0.096
WH ratio		149	-0.037	-0.005	0.086	-0.060		245	0.043	-0.040	-0.026	-0.010
Body fat (%)		149	0.026	0.002	0.033	-0.032		244	0.018	-0.055	0.015	-0.070
Grip (kg)		148	-0.065	0.076	0.019	0.070		244	0.005	0.039	-0.065	0.109
Back (kg)		151	-0.065	0.099	0.001	0.106		241	-0.017	0.069	-0.015	0.093
Jump (cm)		151	0.025	0.084	-0.056	0.124		241	-0.109	0.120	0.081	0.078
Push-ups (rep/min)		139	0.084	-0.102	-0.097	-0.062		229	-0.063	0.031	0.044	-0.003
Sit-ups (rep/min)		139	0.102	-0.067	-0.115	0.004		218	-.133*	0.119	0.122	0.030
Balance (s)		151	-0.061	0.092	0.037	0.062		246	-0.082	0.117	0.041	0.099
Flexibility (cm)		148	-0.016	-0.009	-0.012	0.006		245	-0.031	0.052	-0.023	0.114
Reaction time (s)		146	0.102	-0.078	-0.064	-0.055		245	0.062	-0.063	-0.088	0.029
VO_2_ max (mL/(kg×min))		114	-0.046	0.030	0.039	0.030		192	-0.112	0.103	0.088	0.022

*BMI* Body mass index, the weight in kilograms divided by the square of the height in meters, *WC* Waist circumference, *HC* Hip circumference, *WH ratio* Waist/hip circumference ratio, *ID* Inhalation duration, *ED* Exhalation duration, *I/E ratio* Inhalation exhalation ratio, *VO*_*2*_* max* The maximum rate of oxygen consumption, *N* Newton, *kg* Kilogram, *cm* Centimeter, *ml* Milliliter. Age^1^, n^1^, RR^1^,ID^1^, ED^1^, and I/E ratio^1^ are for the young group (20-39 years), while age^2^, n^2^, RR^2^, ID^2^, ED^2^, and I/E ratio^2^ for the middle-aged group (40-59 years). * *p* < 0.05

### The comparison of physical fitness components in lower/higher respiratory parameter groups within separate age groups of men and women

For further exploration of the link between respiration patterns and physical fitness, each parameter (RR, ID, ED, and I/E ratio) was divided into two groups using the median split method. This division was performed to compare physical fitness between the lower and higher respiratory parameter groups. For men, the comparison results indicated that there were no significant differences between the two groups in terms of age, height, weight, BMI, waist circumference, hip circumference, and waist-hip circumference ratio. However, as shown in Table 5 (only significant results are presented), men with lower RRs had a significantly faster reaction in two age groups. In addition, middle-aged men with lower RRs jumped higher. Men with longer EDs had shorter reaction times in the young group and jumped higher in the middle-aged group. Men with longer IDs jumped higher in both age groups. There was no significant difference between higher and lower I/E ratios in men.

**Table 5:** The comparison of physical fitness in lower/higher respiratory parameter groups within two age groups of men (*n*=163)

**Age** **group**	**Respiratory Parameter (RP)**	**Physical** **fitness**	**Lower RP**	**n**^**1**^	**Higher RP**	**n**^**2**^	**z**	***p***	**r**
20-39	**RR**	**Reaction time**	**0.50 (0.07)**	**36**	**0.55 (0.10)**	**32**	**3.23**	**0.001**	**0.39**
	ID	Vertical Jump	32.8 (13.2)	35	38.9 (14.3)	35	1.63	0.102	0.20
	ED	Reaction time	0.54 (0.09)	34	0.50 (0.07)	34	2.69	0.007	0.33
40-59	**RR**	**Reaction time**	**0.56 (0.12)**	**43**	**0.61 (0.11)**	**42**	**2.52**	**0.012**	**0.27**
		**Vertical Jump**	**30.1 (12.7)**	**47**	**24.6 (10.1)**	**46**	**3.10**	**0.002**	**0.32**
	ID	Vertical Jump	25.2 (11.8)	47	29.0 (13.5)	46	2.19	0.028	0.23
		Flexibility	1.95 (12.9)	44	6.80 (17.0)	45	2.00	0.045	0.21
	ED	Vertical Jump	25.3 (11.5)	46	29.1 (12.6)	47	2.38	0.017	0.25

*RR* Respiration rate, *ID* Inhalation duration, *ED* Exhalation duration, r Effect size. N1 represents the number of participants with lower value of the respiratory parameters, while n2 for the higher value of the respiratory parameters

Regarding the respiratory parameters in women, there were no significant differences in the physical fitness components between the groups with lower and higher respiratory parameters. Consequently, these results are not presented.

### The comparison of physical fitness components in groups based on inhalation duration and exhalation duration within separate age groups of men and women

For male participants, the cluster analysis identified two sub-groups based on ID and ED. The first cluster had relatively longer ID and ED, whereas the second had shorter ID and ED in both young and middle-aged groups. Table 6 shows the comparison results between these two clusters. The RR was significantly lower in the longer ID and ED cluster for both young and middle-aged men (large effect size). In the longer ID and ED cluster, the reaction time was shorter (medium effect size) in both age groups, while the vertical jump was higher (small effect size) only in the middle-aged group.

**Table 6:** The comparison of the parameters in groups based on inhalation and exhalation duration within two age groups of men (*n*=163)

**Age group**	**ALL parameters (men)**	**Longer ID and ED**		**Shorter ID and ED**		**z**	**p**	**r**
		n	Median (IQR)	n	Median (IQR)			
**20-39**	RR	36	13.8 (2.75)	34	18.3 (2.27)	-6.99	0.000	0.84
	ID	36	1.81 (0.61)	34	1.29 (0.37)	-6.13	0.000	0.73
	ED	36	2.56 (0.57)	34	1.86 (0.34)	-6.85	0.000	0.82
	I/E ratio	36	0.69 (0.27)	34	0.73 (0.20)	-0.16	0.869	0.02
	Age (year)	36	31.0 (9.75)	34	33.0 (9.50)	-0.62	0.533	0.07
	Height (cm)	36	176 (9.15)	34	176 (7.45)	-0.06	0.948	0.01
	Weight (kg)	36	82.4 (16.5)	34	76.1 (24.0)	-0.28	0.778	0.03
	BMI (kg/m^2^)	36	25.8 (4.99)	34	25.3 (5.97)	-0.04	0.972	0.00
	WC (cm)	36	91.8 (11.8)	31	87.2 (16.4)	-0.84	0.403	0.10
	HC (cm)	36	102 (7.78)	31	100 (14.8)	-0.87	0.382	0.11
	WH ratio	36	0.89 (0.08)	31	0.86 (0.10)	-0.52	0.606	0.06
	Body fat (%)	36	25.5 (6.67)	31	24.3 (7.70)	-0.35	0.725	0.04
	Grip (kg)	36	43.9 (10.8)	33	43.5 (13.1)	-1.17	0.244	0.14
	Back (kg)	36	127 (34.8)	33	115 (42.5)	-1.93	0.053	0.23
	Jump (cm)	36	39.1 (16.6)	34	34.4 (10.3)	-1.96	0.050	0.23
	Push-ups (rep/min)	36	20.0 (19.5)	29	20.0 (20.0)	-0.42	0.677	0.05
	Sit-ups (rep/min)	36	27.5 (11.5)	31	28.0 (14.0)	-1.14	0.254	0.14
	Balance (s)	36	17.8 (24.4)	34	13.6 (17.2)	-0.56	0.573	0.07
	Flexibility (cm)	36	4.90 (16.0)	33	0.50 (12.7)	-1.44	0.151	0.17
	**Reaction time (s)**	**36**	**0.50 (0.07)**	**32**	**0.55 (0.08)**	**-2.76**	**0.006**	**0.33**
	VO_2_ max (mL/(kg×min))	26	38.6 (12.0)	18	40.9 (9.48)	-0.47	0.642	0.07
**40-59**	RR	34	12.6 (3.19)	59	18.5 (3.29)	-7.94	0.000	0.82
	ID	34	2.03 (0.59)	59	1.27 (0.25)	-7.13	0.000	0.74
	ED	34	2.71 (0.58)	59	1.89 (0.32)	-7.45	0.000	0.77
	I/E ratio	34	0.78 (0.30)	59	0.70 (0.15)	-1.55	0.122	0.16
	Age (year)	34	46.5 (10.5)	59	51.0 (10.0)	-1.64	0.101	0.17
	Height (cm)	34	171 (10)	59	173 (7)	-1.12	0.261	0.12
	Weight (kg)	34	73.0 (13.9)	59	75.5 (15.5)	-0.99	0.321	0.10
	BMI (kg/m^2^)	33	25.3 (3.46)	59	25.2 (5.19)	-0.44	0.661	0.05
	WC (cm)	33	89.1 (9.70)	57	92.4 (11.7)	-1.17	0.241	0.12
	HC (cm)	33	96.9 (9.45)	57	98.3 (9.95)	-0.49	0.627	0.05
	WH ratio	34	0.92 (0.07)	57	0.93 (0.09)	-1.40	0.161	0.15
	Body fat (%)	34	23.5 (6.53)	57	24.8 (7.78)	-0.78	0.436	0.08
	Grip (kg)	34	43.1 (8.78)	59	40.5 (9.30)	-1.43	0.152	0.15
	Back (kg)	34	118 (36.9)	59	104 (42.5)	-1.44	0.151	0.15
	**Jump (cm)**	**34**	**30.7 (11.0)**	**59**	**25.2 (11.3)**	**-3.20**	**0.001**	**0.33**
	Push-ups (rep/min)	33	20.0 (16.0)	46	16.5 (16.0)	-0.57	0.571	0.06
	Sit-ups (rep/min)	31	21.0 (18.0)	44	20.0 (11.8)	-1.34	0.180	0.15
	Balance (s)	33	12.7 (14.1)	57	8.50 (11.7)	-1.03	0.305	0.11
	Flexibility (cm)	34	6.10 (17.4)	55	2.70 (14.5)	-1.16	0.247	0.12
	**Reaction time (s)**	**31**	**0.55 (0.12)**	**54**	**0.60 (0.13)**	**-2.52**	**0.012**	**0.27**
	VO_2_ max (mL/(kg×min))	21	39.7 (11.7)	31	40.6 (13.8)	-0.45	0.654	0.06

*BMI* Body mass index, the weight in kilograms divided by the square of the height in meters, *WC* Waist circumference, *HC* Hip circumference, *WH ratio* Waist/hip circumference ratio, *ID* Inhalation duration, *ED* Exhalation duration, *I/E ratio* Inhalation exhalation ratio, *VO*_*2*_* max* The maximum rate of oxygen consumption, *N* Newton, *kg* Kilogram, *cm* Centimeter, *ml* Milliliter

The cluster analysis also identified two sub-groups in women based on ID and ED. The first cluster had relatively longer ID and ED, and the second had shorter ID and ED. As shown in Table 7, women from both age groups exhibited significantly lower RR in the cluster with longer ID and ED, and middle-aged women in the longer ID and ED cluster had a higher number of sit-ups in one minute and better cardiopulmonary endurance with small effect size.

**Table 7:** The comparison of the parameters in groups based on inhalation and exhalation duration within two age groups of women (*n*=401)

**Age group**	ALL parameters (women)	**Longer ID and ED**		**Shorter ID and ED**		Z	P	r
		n1	Median (IQR)	n2	Median (IQR)			
**20**−**39**	RR	52	14.2 (3.05)	100	18.5 (2.93)	-9.91	0.000	0.80
	ID	52	1.72 (0.33)	100	1.23 (0.29)	-9.28	0.000	0.75
	ED	52	2.51 (0.59)	100	1.91 (0.35)	-8.88	0.000	0.72
	I/E ratio	52	0.70 (0.19)	100	0.67 (0.18)	-1.15	0.250	0.09
	Age (year)	52	34.0 (5.00)	100	34.0 (9.00)	-0.62	0.538	0.05
	Height (cm)	52	1.61 (0.08)	100	1.61 (0.07)	-0.14	0.889	0.01
	Weight (kg)	52	57.8 (16.2)	100	58.6 (13.4)	-0.49	0.623	0.04
	BMI (kg/m^2^)	52	22.5 (6.38)	100	22.0 (5.03)	-0.07	0.941	0.01
	WC (cm)	51	73.2 (14.8)	98	73.4 (13.6)	-0.80	0.422	0.07
	HC (cm)	51	93.7 (11.5)	98	93.8 (9.35)	-0.78	0.435	0.06
	WH ratio	51	0.79 (0.07)	98	0.79 (0.08)	-0.16	0.873	0.01
	Body fat (%)	52	27.5 (9.75)	97	27.6 (8.88)	-0.61	0.542	0.05
	Grip (kg)	51	25.0 (6.30)	97	24.5 (6.75)	-0.50	0.620	0.04
	Back (kg)	51	61.5 (20.8)	100	63.2 (23.6)	-0.64	0.524	0.05
	Jump (cm)	52	22.9 (3.65)	99	22.0 (8.00)	-0.21	0.837	0.02
	Push-ups (rep/min)	52	15.5 (15.0)	87	17.0 (13.0)	-0.44	0.657	0.04
	Sit-ups (rep/min)	51	22.0 (12.0)	88	23.5 (14.8)	-0.66	0.509	0.06
	Balance (s)	52	27.5 (29.8)	99	20.5 (26.4)	-0.91	0.360	0.07
	Flexibility (cm)	52	8.45 (8.48)	96	9.05 (17.2)	-0.16	0.869	0.01
	Reaction time (s)	51	0.56 (0.08)	95	0.57 (0.10)	-1.11	0.269	0.09
	VO_2_ max (mL/(kg×min))	38	43.5 (16.1)	76	40.0 (10.6)	-1.16	0.245	0.11
**40**−**59**	RR	93	13.5 (3.11)	154	18.8 (3.13)	-12.94	0.000	0.82
	ID	93	1.71 (0.51)	154	1.23 (0.22)	-12.28	0.000	0.78
	ED	93	2.68 (0.66)	154	1.91 (0.43)	-11.53	0.000	0.73
	I/E ratio	93	0.67 (0.25)	154	0.65 (0.16)	-1.27	0.205	0.08
	Age (year)	93	47.0 (10.5)	154	48.0 (10.0)	-1.16	0.245	0.07
	Height (cm)	93	1.62 (0.06)	154	1.61 (0.07)	-1.53	0.127	0.10
	Weight (kg)	93	61.0 (12.5)	154	61.3 (11.8)	-0.16	0.871	0.01
	BMI (kg/m^2^)	93	22.4 (4.00)	154	23.5 (4.01)	-1.22	0.222	0.08
	WC (cm)	92	76.0 (11.6)	153	79.2 (12.3)	-1.13	0.258	0.07
	HC (cm)	92	94.5 (10.1)	153	94.6 (8.30)	-0.26	0.798	0.02
	WH ratio	92	0.81 (0.09)	153	0.83 (0.08)	-1.20	0.232	0.08
	Body fat (%)	92	29.2 (6.61)	152	30.2 (6.81)	-0.68	0.499	0.04
	Grip (kg)	92	25.5 (7.20)	152	25.8 (7.63)	-0.27	0.785	0.02
	Back (kg)	91	66.6 (25.0)	150	66.4 (22.0)	-0.14	0.889	0.01
	Jump (cm)	92	20.1 (6.15)	151	18.6 (7.00)	-1.84	0.065	0.12
	Push-ups (rep/min)	90	15.0 (16.0)	139	15.0 (13.0)	-0.81	0.417	0.05
	**Sit-ups (rep/min)**	**81**	**20.0 (15.5)**	**137**	**16.0 (10.5)**	**-2.19**	**0.028**	**0.15**
	Balance (s)	93	18.9 (26.5)	153	14.5 (16.2)	-1.90	0.057	0.12
	Flexibility (cm)	92	11.4 (13.4)	153	10.1 (12.6)	-1.22	0.221	0.08
	Reaction time (s)	92	0.59 (0.10)	153	0.60 (0.11)	-0.91	0.365	0.06
	**VO**_**2**_** max (mL/(kg×min))**	**68**	**36.2 (7.35)**	**124**	**33.8 (7.35)**	**-2.21**	**0.027**	**0.16**

*BMI* Body mass index, the weight in kilograms divided by the square of the height in meters, *WC* Waist circumference, *HC* Hip circumference, *WH ratio* waist/hip circumference ratio, *ID* Inhalation duration, *ED* Exhalation duration, *I/E ratio* Inhalation exhalation ratio, *VO*_*2*_* max* The maximum rate of oxygen consumption, *N* Newton, *kg* Kilogram, *cm* Centimeter, *ml* Milliliter

## Discussion

To the best of our knowledge, this is the first study that examined the links between respiratory patterns (RR and I/E ratio) during spontaneous breathing and the most physical fitness components (body size, body composition, muscle strength, muscle endurance, balance, flexibility, visuomotor reaction time, and cardiopulmonary endurance). The main findings indicated that RR and the I/E ratio were not substantially correlated with all physical fitness components in women. In contrast, men with lower RRs exhibited significantly shorter visuomotor reaction times in both the young and middle-aged groups, and demonstrated significantly higher vertical jump heights in the middle-aged group.

Visuomotor reaction time relies on the intact functioning of sensory systems, cognitive processing, and motor performance, and it serves as a valuable indicator of an individual's sensorimotor coordination and overall performance [43] while also being linked to factors such as arousal and attention. Kovacs et al. conducted a study revealing that increased arousal, induced by mental stress, significantly increased the reaction time [44]. Furthermore, attention was found to be closely linked to reaction time, with higher levels of attention resulting in shorter reaction times [45, 46]. Our findings revealed that men with lower respiration rates exhibited faster visuomotor reaction speeds, potentially suggesting that men with lower RR (13.8 (2.75) for ages 20-39 and 12.6 (3.19) for ages 40-59) have better visual motor coordination, higher attention levels, and/or experience lower mental stress than men with higher RR (18.3 (2.27) for ages 20-39 and 18.5 (3.29) for age 40-59). We have not found any other studies to verify this result. However, numerous studies have found that voluntarily decreasing RR increases ease, comfort, relaxation, and positive energy while reducing anxiety, dejection, anger, hostility, and confusion [8]. In addition, Krzysztof et al. divided participants into three tertiles based on their spontaneous respiration rate (10.6 breaths/min in the first tertile, 14.8 breaths/min in the second tertile, and 18.0 breaths/min in the third tertile), and they discovered that participants from the third tertile, characterized by a faster respiratory rate, exhibited greater sympathetic activity compared to subjects from the first tertile (*P* < 0.001, Hedges' g = 4.7) [47]. Furthermore, the executive function was improved after voluntarily decreasing the breathing rate to 6 reps/min compared to natural breathing, with higher scores observed for Stroop interference accuracy [48–50].

In addition to the results of reaction time, men with lower RR had significantly higher jump height but not on other muscular performance. The countermovement jump was tested in the present study, which requires a lower limb explosive power [51, 52], as well as refined, muscular coordination as it requires the activation of stretch reflex (or myotatic reflex, muscle stretch-shortening cycle) on the legs and arms [53, 54]. RR and tidal volume are negatively correlated [22], and the diaphragm muscle takes 70-80% work for tidal breathing [55], which means a healthy person with lower RR should have greater activation of the diaphragm muscle than those with higher RR. The stability of the trunk is the basis of all functional movements [56]. The diaphragm muscle is one of the main core muscles for trunk stability [23], as it works to control intra-abdominal pressure and reduce stress on the spine through cooperation with the abdominal and pelvic floor muscles [57]. Therefore, we suggest that people with lower RR have a better function of the diaphragm that optimizes core stability, facilitates body coordination, and results in a better countermovement jump. Stress level might also contribute to the results, as Melanie and Vanessa summarized in their paper that acute and chronic stress have both been found and suggested to affect motor functioning directly but also indirectly in everyday motor task due to complex links between changes in hormonal, (neuro-)physiological, psychological, cognitive, and motor functions [58].

One finding that has puzzled us is the unsubstantial difference in reaction time between women with slower breathing rates and those with faster breathing rates. So far, we do not have an explanation for this finding, and this requires further investigation.

Another perplexing finding is that individuals with different I/E ratios did not exhibit substantial differences in physical fitness. Inhalation is primarily influenced by sympathetic activity, while exhalation is predominantly associated with parasympathetic activity. Lower I/E ratios are often indicative of a higher level of relaxation. However, despite these associations, differing I/E ratios did not yield distinguishable effects on physical fitness. This observation raises the possibility that I/E ratios may not hold as much influence over physical fitness outcomes.

### Limitations

The present study had some limitations. At first, the sample size was unbalanced between ages and genders. The female participants outnumbered the males and a larger proportion of middle-aged participants compared to young participants. Secondly, many male participants were not willing to have their push-ups and cardiopulmonary endurance tested, and the sample size of young men did not meet the estimated number. Third, we did not collect information regarding the participants' smoking status, medical history, daily level of physical activity, and exposure to polluted environments, all of which could be factors influencing their respiratory patterns and physical fitness. Fourth, the current study only investigated young and middle-aged people; further study on elders is necessary.

### Practical suggestions

Visuomotor reaction time was associated with increased injury risk [59], and visuomotor reaction time may be a potential target for prevention and rehabilitation strategies in individuals with ankle sprains [60]. Therefore, physiotherapists, physical education teachers, and coaches should consider the respiration rates of their clients, students, or athletes in order to enhance reaction speed, improve motor coordination, and prevent injuries.

## Conclusion

Women’s respiratory patterns (RR and I/E ratio) were not substantially correlated with physical fitness. Men with lower RRs may have better visual-motor coordination and/or sustained attention, while middle-aged men with lower RRs may also have greater leg explosive power and neuromuscular coordination. Future studies may explore methods, such as breathing or physical exercises, to reduce spontaneous RR in men with relatively high RRs. The I/E ratios were not significantly correlated with physical fitness in young men, while the relationship between middle-aged men’s I/E ratios and their physical fitness warrants further investigation.

## Data Availability

The data that support the findings of this study are available on request from the corresponding author Jing Xiao.

## References

[1] Fonseca Del Pozo FJ, Alonso JV, Álvarez MV, Orr S, Cantarero FJL (2017). Physical fitness as an indicator of health status and its relationship to academic performance during the prepubertal period. Health Promot Perspect.

[2] Fahey T, Insel P, Roth W (2018). Fit & Well: Core Concepts and Labs in Physical Fitness and Wellness.

[3] Russo MA, Santarelli DM, O’Rourke D (2017). The physiological effects of slow breathing in the healthy human. Breathe (Sheff).

[4] Sakamoto A, Naito H, Chow C-M (2014). Hyperventilation as a strategy for improved repeated sprint performance. J Strength Cond Res.

[5] Jacob C, Keyrouz C, Bideau N, Nicolas G, El Hage R, Bideau B (2015). Pre-exercise hyperventilation can significantly increase performance in the 50-meter front crawl. Sci Sports.

[6] Benchetrit G (2000). Breathing pattern in humans: diversity and individuality. Respir Physiol.

[7] Nicolò A, Massaroni C, Schena E, Sacchetti M (2020). The Importance of Respiratory Rate Monitoring: From Healthcare to Sport and Exercise. Sensors (Basel).

[8] Zaccaro A, Piarulli A, Laurino M, Garbella E, Menicucci D, Neri B (2018). How breath-control can change your life: a systematic review on psycho-physiological correlates of slow breathing. Front Hum Neurosci.

[9] Bernardi L, Spadacini G, Bellwon J, Hajric R, Roskamm H, Frey AW (1998). Effect of breathing rate on oxygen saturation and exercise performance in chronic heart failure. Lancet.

[10] Ublosakka-Jones C, Tongdee P, Pachirat O, Jones DA (2018). Slow loaded breathing training improves blood pressure, lung capacity and arm exercise endurance for older people with treated and stable isolated systolic hypertension. Exp Gerontol.

[11] Manandhar SA, Pramanik T (2019). Immediate effect of slow deep breathing exercise on blood pressure and reaction time. Mymensingh Med J.

[12] Barrett K, Barman S, Yuan J, Brooks H. Ganong’s Review of Medical Physiology, Twenty Sixth Edition. 26th edition. New York: McGraw Hill / Medical; 2019.

[13] Lu Y, Yi L, Liu D, Li J, Sun L, Zhang Z (2012). Alkalosis leads to the over-activity of cortical principal neurons. Neurosci Lett.

[14] Tombaugh GC, Somjen GG (1996). Effects of extracellular pH on voltage-gated Na+, K+ and Ca2+ currents in isolated rat CA1 neurons. J Physiol.

[15] Lewis T (1908). Studies of the relationship between respiration and blood-pressure: Part II. Facts bearing on the relationship of different factors in the production of respiratory curves of blood-pressure. J Physiol..

[16] Gerritsen RJS, Band GPH (2018). Breath of life: the respiratory vagal stimulation model of contemplative activity. Front Hum Neurosci.

[17] Carroll RG. 9 - Integrated Cardiovascular Function. In: Carroll RG, editor. Elsevier’s Integrated Physiology. Philadelphia: Mosby; 2007. p. 91–8. Cited 2023 Jul 4. Available from: https://www.sciencedirect.com/science/article/pii/B9780323043182500157.

[18] Armstrong M, Kerndt CC, Moore RA. Physiology, Baroreceptors. StatPearls. Treasure Island (FL): StatPearls Publishing; 2023. Cited 2023 Jul 4. Available from: http://www.ncbi.nlm.nih.gov/books/NBK538172/. 30844199

[19] Bae D, Matthews JJL, Chen JJ, Mah L (2021). Increased exhalation to inhalation ratio during breathing enhances high-frequency heart rate variability in healthy adults. Psychophysiology.

[20] Van Diest I, Verstappen K, Aubert AE, Widjaja D, Vansteenwegen D, Vlemincx E (2014). Inhalation/Exhalation ratio modulates the effect of slow breathing on heart rate variability and relaxation. Appl Psychophysiol Biofeedback.

[21] Ratnovsky A, Elad D (2005). Anatomical model of the human trunk for analysis of respiratory muscles mechanics. Respir Physiol Neurobiol.

[22] Wu H, Xiao W, Xu X, Gu Y, Lu F, Shi J (2015). Relationship of tidal volume to peak flow, breath rate, I: E and plateau time: mock study. Am J Med Sci.

[23] Huxel Bliven KC, Anderson BE (2013). Core stability training for injury prevention. Sports Health.

[24] Horii O, Sasaki M (2023). Influences of trunk stability on exercise performance of closed kinetic chain of upper and lower limbs. J Phys Ther Sci.

[25] Saeterbakken AH, Stien N, Andersen V, Scott S, Cumming KT, Behm DG (2022). The Effects of Trunk Muscle Training on Physical Fitness and Sport-Specific Performance in Young and Adult Athletes: A Systematic Review and Meta-Analysis. Sports Med.

[26] Katayıfçı N, Boşnak Güçlü M, Şen F (2022). A comparison of the effects of inspiratory muscle strength and endurance training on exercise capacity, respiratory muscle strength and endurance, and quality of life in pacemaker patients with heart failure: a randomized study. Heart Lung.

[27] Chang Y-C, Chang H-Y, Ho C-C, Lee P-F, Chou Y-C, Tsai M-W (2021). Effects of 4-Week Inspiratory Muscle Training on Sport Performance in College 800-Meter Track Runners. Medicina (Kaunas).

[28] Bennett J, Munavvar M, Walker P, Phillips G (2020). Respiratory advice for the non-respiratory physician in the time of COVID-19. Clin Med (Lond).

[29] Liu Q, Liu Y, Leng X, Han J, Xia F, Chen H (2020). Impact of chronic stress on attention control: evidence from behavioral and event-related potential analyses. Neurosci Bull.

[30] Ross R, Mongan WM, O’Neill P, Rasheed I, Fontecchio A, Dion G (2021). An Adaptively Parameterized Algorithm Estimating Respiratory Rate from a Passive Wearable RFID Smart Garment. Proc COMPSAC.

[31] Kocjan J, Gzik-Zroska B, Nowakowska K, Burkacki M, Suchoń S, Michnik R (2018). Impact of diaphragm function parameters on balance maintenance. PLoS One.

[32] Teixeira-Salmela LF, Parreira VF, Britto RR, Brant TC, Inácio EP, Alcântara TO (2005). Respiratory pressures and thoracoabdominal motion in community-dwelling chronic stroke survivors. Arch Phys Med Rehabil.

[33] Szczygieł E, Blaut J, Zielonka-Pycka K, Tomaszewski K, Golec J, Czechowska D (2018). The impact of deep muscle training on the quality of posture and breathing. J Mot Behav.

[34] Zhang Y, He Z, Xu J (2017). National Physical Fitness Surveillance and Evaluation.

[35] Liang W-M, Bai Z-M, Aihemaiti M, Yuan L, Hong Z-M, Xiao J (2022). Women’s respiratory movements during spontaneous breathing and physical fitness: a cross-sectional, correlational study. Int J Environ Res Public Health.

[36] Garatachea N, Cavalcanti E, García-López D, González-Gallego J, de Paz JA (2007). Estimation of energy expenditure in healthy adults from the YMCA submaximal cycle ergometer test. Eval Health Prof.

[37] Jaric S (2002). Muscle strength testing: use of normalisation for body size. Sports Med.

[38] Markovic G, Jaric S (2004). Movement performance and body size: the relationship for different groups of tests. Eur J Appl Physiol.

[39] Kang H (2021). Sample size determination and power analysis using the G*Power software. J Educ Eval Health Prof.

[40] Prion S, Haerling KA (2014). Making Sense of Methods and Measurement: Spearman-Rho Ranked-Order Correlation Coefficient. ClinSimul Nurs.

[41] Fritz CO, Morris PE, Richler JJ (2012). Effect size estimates: current use, calculations, and interpretation. J Exp Psychol Gen.

[42] Park J-S, Hwang N-K, Kim H-H, Choi J-B, Chang M-Y, Jung Y-J (2019). Effects of lingual strength training on oropharyngeal muscles in South Korean adults. J Oral Rehabil.

[43] Balakrishnan G, Uppinakudru G, Girwar Singh G, Bangera S, Dutt Raghavendra A, Thangavel D (2014). A comparative study on visual choice reaction time for different colors in females. Neurol Res Int.

[44] Kovacs C, Bories T (2010). Effects of increased physiological arousal on upper extremity reaction and movement times in healthy young adults. Neurosci Int.

[45] Karwowski W, editor. International Encyclopedia of Ergonomics and Human Factors - 3 Volume Set, 2nd edition. Boca Raton: CRC Press; 2006.

[46] Golmohammadi R, Yousefi H, Safarpour Khotbesara N, Nasrolahi A, Kurd N (2021). Effects of light on attention and reaction time: a systematic review. J Res Health Sci.

[47] Narkiewicz K, van de Borne P, Montano N, Hering D, Kara T, Somers VK (2006). Sympathetic neural outflow and chemoreflex sensitivity are related to spontaneous breathing rate in normal men. Hypertension.

[48] Laborde S, Allen MS, Borges U, Hosang TJ, Furley P, Mosley E (2022). The influence of slow-paced breathing on executive function. J Psychophysiol.

[49] Liang W-M, Xiao J, Ren F-F, Chen Z-S, Li C-R, Bai Z-M (2023). Acute effect of breathing exercises on muscle tension and executive function under psychological stress. Front Psychol.

[50] Prinsloo GE, Rauch HGL, Lambert MI, Muench F, Noakes TD, Derman WE (2011). The effect of short duration heart rate variability (HRV) biofeedback on cognitive performance during laboratory induced cognitive stress. Appl Cogn Psychol.

[51] Markovic G, Dizdar D, Jukic I, Cardinale M (2004). Reliability and factorial validity of squat and countermovement jump tests. J Strength Cond Res.

[52] Martinez N, Campbell B, Franek M, Buchanan L, Colquhoun R (2016). The effect of acute pre-workout supplementation on power and strength performance. J Int Soc Sports Nutr.

[53] Król H, Mynarski W (2012). A comparison of mechanical parameters between the counter movement jump and drop jump in biathletes. J Hum Kinet.

[54] Petrigna L, Karsten B, Marcolin G, Paoli A, D’Antona G, Palma A, et al. A Review of Countermovement and Squat Jump Testing Methods in the Context of Public Health Examination in Adolescence: Reliability and Feasibility of Current Testing Procedures. Frontiers in Physiology. 2019;10. Cited 2023 Jul 3. Available from: https://www.frontiersin.org/articles/10.3389/fphys.2019.01384. 10.3389/fphys.2019.01384 PMC6853898 31787902

[55] Essentials of Kinesiology for the Physical Therapist Assistant - 3rd Edition. Cited 2023 Jul 3. Available from: https://shop.elsevier.com/books/essentials-of-kinesiology-for-the-physical-therapist-assistant/mansfield/978-0-323-54498-6.

[56] Cha HG (2018). Effects of trunk stabilization exercise on the local muscle activity and balance ability of normal subjects. J Phys Ther Sci.

[57] Kim E, Lee H (2013). The effects of deep abdominal muscle strengthening exercises on respiratory function and lumbar stability. J Phys Ther Sci.

[58] Krüger M, Lux V (2023). Failure of motor function-A Developmental Embodiment Research perspective on the systemic effects of stress. Front Hum Neurosci.

[59] Brinkman C, Baez SE, Quintana C, Andrews ML, Heebner NR, Hoch MC (2020). The reliability of an upper- and lower-extremity visuomotor reaction time task. J Sport Rehabil.

[60] Song K, Hoch JM, Quintana C, Heebner NR, Hoch MC (2023). Slower visuomotor reaction time in division-I collegiate athletes with a history of ankle sprain. Res Sports Med.

